# Pan-cancer analysis identifies LIFR as a prognostic and immunological biomarker for uterine corpus endometrial carcinoma

**DOI:** 10.3389/fonc.2023.1118906

**Published:** 2023-02-28

**Authors:** Fang Zhang, Yali Wang, Hongjuan Li, Li Li, Xiaofeng Yang, Xiaoyan You, Lina Tang

**Affiliations:** ^1^ Department of Obstetrics and Gynecology, Zhengzhou Central Hospital Affiliated to Zhengzhou University, Zhengzhou, China; ^2^ Metabolic Disease Research Center, Zhengzhou Central Hospital Affiliated to Zhengzhou University, Zhengzhou, China

**Keywords:** LIFR, cancer, prognosis, survival, immune infiltration

## Abstract

**Background:**

Leukemia inhibitory factor (LIF) exhibits significant tumor-promoting function, while its cognate receptor (LIFR) is considered to act as either a tumor promoter or suppressor. Dysregulation of LIF and LIFR is associated with the initiation, progression and metastasis of multiple cancer entities. Although increasing numbers of studies are revealing an indispensable critical role of LIFR in tumorigenesis for various different cancers, no systematic analysis of LIFR has appeared thus far.

**Methods:**

Here, we comprehensively analyzed the expression profile and prognostic value of LIFR, and correlations between LIFR and the infiltration of immune cells and clinicopathological parameters across different tumor types using several bioinformatic tools. The expression profile of LIFR in various tumor types and clinical stages was investigated using the TIMER2 and GEPIA2 databases. Genetic alternations of LIFR were extracted from cBioPortal. The prognostic value of LIFR was assessed using GEPIA2 and Sanger box databases, and correlations between LIFR expression and immune infiltration were analyzed using the CIBERSORT method and TIMER2 database. The correlations between LIFR expression and immune and stromal scores were assessed using ESTIMATE. We also analyzed correlations between LIFR and immunoregulators. Finally, we detected an effect of LIFR on Uterine Corpus Endometrial Carcinoma (UCEC) and evaluated the expression level of LIFR in clinical UCEC samples.

**Results:**

Aberrant expression of LIFR in cancers and its prognosis ability, especially in UCEC was documented. Significantly lower levels of LIFR expression level correlated with better prognosis in multiple tumor types. LIFR expression was positively correlated with the abundance of cancer-associated fibroblasts (CAFs) and endothelial cells in the tumor microenvironment. Additionally, LIFR expression was strongly associated with the presence of immune modulators and checkpoint genes. Overexpression of LIFR suppressed the migration and invasion of UCEC cells *in vitro*.

**Conclusion:**

Our pan-cancer detection data provided a novel understanding of the roles of LIFR in oncogenesis.

## Introduction

The initiation, progression and metastasis of cancers have close connections to inflammation and inflammatory cytokines, but the underlying mechanisms are still poorly understood. Given the complexity of tumorigenesis, it is critical to conduct a pan-cancer survey of the expression of any potential gene of interest and to evaluate its clinical prognostic value and potential mechanisms of action. The publicly available functional genomics data available in TCGA (The Cancer Genome Atlas) and the GEO (Gene Expression Omnibus) have contributed to this endeavor in many different cancers.

LIF and LIFR, members of the interleukin-6 (IL-6) cytokine family, constitute a poorly-defined pathway connecting inflammation to cancer (1). Although other IL-6 family members have been shown to regulate the metastasis of multiple cancer types, the role of LIF and LIFR remains challenging to access. LIFR, also known as CD118, is a transmembrane receptor that mediates signal transduction of its corresponding ligands oncostatin M (OSM), LIF, cardiotrophin 1 (CT1) and Ciliary Neurotrophic Factor (CNTF) in multiple pathological conditions mainly in cancer progression and the promotion of metastasis (2). Among the ligands of LIFR, LIF is overexpressed and has been identified playing a tumor-promoting role in various tumors, including prostate, nasopharyngeal, breast, gastric, endometrial, colorectal, melanoma, osteosarcoma, lung and pancreatic cancers (2–10). Recently, increasing numbers of studies have been exploring the cancer type-specific role of LIFR in these cancers (11–16). LIFR is expressed in a variety of organs and cell types and is involved in angiogenesis, cancer progression, development and regulation of stem cells (2, 4, 10, 17). Stromal and epithelial cells secrete LIF that binds the LIFR and activates PI3K/AKT, JAK/STAT1/3, mTORC1/P70S6k, MAPK and Hippo/YAP signaling pathways. Clinically, the activation of the LIF/LIFR axis is correlated with poor prognosis and resistance to anti-cancer therapies (18).

However, current studies have limited the investigation of LIFR to a few tumor types, and the correlation with prognosis and immune parameters remains unclear in most cancers. To explore LIFR expression at the pan-cancer level, we extracted LIFR expression at the gene and protein levels from public databases and evaluated its effects on survival, immune infiltration, immune-related genes, and functional pathways in various different cancers. Our results revealed the function of LIFR as a tumor suppressor in multiple tumors, indicating the low levels of LIFR expression may decrease patient survival rates. Furthermore, we validated a tumor suppressor role of LIFR in UCEC. In summary, LIFR is a promising therapeutic target and prognostic biomarker for many cancer entities.

## Materials and methods

### Expression profile analysis

The expression profile of LIFR between tumor and adjacent normal tissues was detected using TIMER2 (http://timer.cistrome.org/) and the GTEx data was obtained from GEPIA2 (http://gepia2.cancer-pku.cn/#analysis).

The relationship between LIFR expression and different pathological stages for various cancers was analyzed *via* GEPIA2.

The data of the total protein level of LIFR was acquired from UCLCAN web (http://ualcan.path.uab.edu/analysis-prot.html).

### Prognostic analysis

We obtained the OS and DFS significance data of LIFR across all tumor types using “Survival Map” module of GEPIA2. We detected the effect of LIFR expression on OS and DFS in various cancer types. The low-expression and high-expression cohorts was splatted using cutoff-low (50%) and cutoff-high (50%) values as the expression thresholds. The survival plots were explored *via* the “Survival Analysis” module of GEPIA2.

The correlation between LIFR and prognosis including DFI, DSS and PFI was estimated by Sanger box (http://sangerbox.com) (19).

We used Kaplan-Meier analysis to analyze the different survival rates between the high- and low- expression groups to assess the prognostic value of LIFR. After adjusting for age and tumor stage, multivariable Cox regression analysis was used to explore the correlation between LIFR expression and DFI, DSS, and PFI.

### Genetic alteration analysis

The genetic alternation data was collected from cBioPortal tool (https://www.cbioportal.org/) using “Quick Selection” and “TCGA Pan-Cancer Atlas Study” options. Then the mutation type, mutation frequency, and copy number variation were obtained by using “Cancer Type Summary” module. We displayed the mutated site information of LIFR in the schematic diagram of the protein structure or the 3D (Three-dimensional) structure *via* the “Mutations” module. We acquired the data of OS, DFS, and PFS differences for various cancer types with or without LIFR genetic alternation, and generated Kaplan-Meier plots with log-rank *P*-value.

### Tumor immune microenvironment and immune cell infiltration

The correlation between LIFR expression and ESTIMATE score, immune score and the stromal score was evaluated *via* Sangerbox using ESTIMATE.

The correlation between immune infiltration and LIFR expression across various tumor types was analyzed by “Immune-Gene” module of the TIMER2 database. The association of LIFR expression with immune infiltration, such as macrophages, endothelial cells, CAFs, CD4^+^ T cells, and CD8^+^ T cells, was estimated applying the TIMER, QUANTISEQ, MCPCOUNTER, CIBERSORT, CIBERSORT-ABS, XCELL, and EPIC algorithms.

### LIFR-related partners enrichment analysis

The protein-protein interaction network was conducted by STRING (https://string-db.org/) setting the following main parameters: meaning of network edges (“evidence”), active interaction sources (“experiments”), minimum required interaction score [“Low confidence (0.150)”], and max number of interactors to show (“no more than 50 interactors” in 1st shell).

The top 100 LIFR-correlated targeting genes were obtained using the “Similar Gene Detection” module of GEPIA2. Then we performed a pairwise gene Pearson correlation analysis of LIFR and selected genes. Furthermore, we generated the heatmap data of the selected genes using “Gene-Corr” module of TIMER2.

We combined the LIFR-binding and LIFR-correlated genes to performed KEGG pathway analysis using Sanger box database.

### UCEC samples collection and western blot assay

UCEC samples were obtained from the Department of Obstetrics and Gynecology in the Zhengzhou Central Hospital. The use of tumor excisions was consented to by the Medical Ethics Committee of Zhengzhou Central Hospital. LIFR antibody was obtained from the Proteintech company (22779-1-AP).

### CCK8 proliferation assay

LIFR stable overexpressed UCEC cell line Ishikawa were seeded in 96‐well plates for cell viability assay. CCK8 reagent was added to incubate at 37°C for 2h. The data was calculated according to the reagent instructions. The absorbance of each sample was measured at 450 nm. The CCK8 proliferation assay have been described previously in detail (20).

### Cell migration and invasion assays

For Transwell migration assay, LIFR stable overexpressed UCEC cell line Ishikawa were separately placed in the top chamber of transwell chambers (8‐μm BioCoat Control Inserts, Corning Costar). The lower chamber was filled with 500 μl DMEM supplemented with 10% FBS. After 24 hours incubation at 37°C, the cells were fixed and stained. The cells in the top chambers were removed and counted. For invasion assay, cells were plated in the matrigel‐coated chamber and the migration assay was performed. The cell migration and invasion assays has been described previously in detail (21).

## Results

### The differential expression of LIFR between normal and tumor tissues

**Figure 1:**
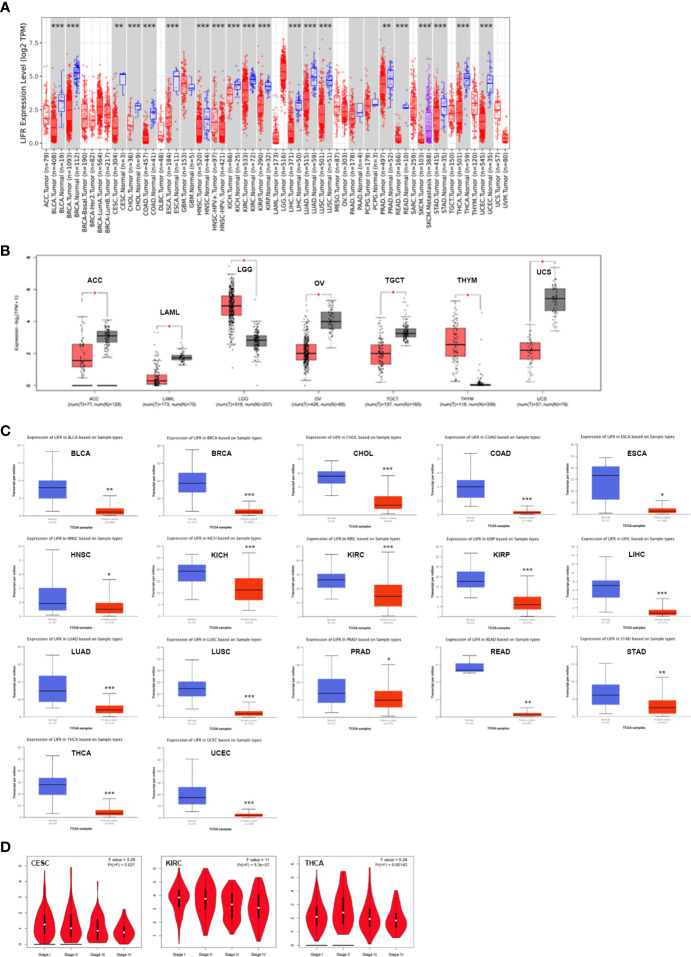
The expression profile of LIFR in different tumors and pathological stages. **(A)** LIFR gene expression status in adjacent normal and tumor tissues across various tumor types in TCGA cohorts as analyzed *via* TIMER2. LIFR was downregulated in BLCA, BRCA, CESC, CHOL, COAD, ESCA, HNSC, KICH, KIRC, KIRP, LIHC, LUAD, LUSC, PRAD, READ, STAD, THCA, and UCEC. *p <.05; **p <.01; ***p <.001. **(B)** LIFR expression in tumor tissues from TCGA and normal tissues in ACC, LAML, LGG, OV, TGCT, THYM and UCS from GTEx cohorts through GEPIA2 *p <.05; **p <.01; ***p <.001. **(C)** Total protein level of LIFR in normal and tumor tissues in BLCA, BRCA, CHOL, COAD, ESCA, HNSC, KICH, KIRC, KIRP, LIHC, LUAD, LUSC, PRAD, READ, STAD, THCA and UCEC. ***p <.001. **(D)** Expression level of LIFR was analyzed in main pathological stages (stage I, stage II, stage III, and stage IV) of CESC and KIRC. Log2 (TPM + 1) was applied for log-scale.

To identify the possible role of LIFR in carcinogenesis, we first assessed the differential expression of LIFR in different cells from normal and tumor tissues. LIFR showed the highest expression in midbrain, followed by basal ganglia and thalamus, with low tissue specificity (**Figure S1A**), while low expression of LIFR was detected in most tumor cell lines, with higher expression in AF22 (brain), SCLC-21H (lung) and HHSteC lines (mesenchymal) (**Figure S1B**). Next, we analyzed LIFR expression across 33 tumor types in TCGA *via* the TIMER database. As shown in **Figure 1A**, compared with normal tissues, we observed significant downregulation of LIFR in BLCA (Bladder Urothelial Carcinoma), BRCA (Breast invasive carcinoma), CESC (Cervical squamous cell carcinoma and endocervical adenocarcinoma), CHOL (Cholangiocarcinoma), COAD (Colon adenocarcinoma), ESCA (Esophageal carcinoma), HNSC (Head and Neck squamous cell carcinoma), KICH (Kidney Chromophobe), KIRC (Kidney renal clear cell carcinoma), KIRP (Kidney renal papillary cell carcinoma), LIHC (Liver hepatocellular carcinoma), LUAD (Lung adenocarcinoma), LUSC (Lung squamous cell carcinoma), PRAD (Prostate adenocarcinoma), READ (Rectum adenocarcinoma), STAD (Stomach adenocarcinoma), THCA (Thyroid carcinoma), and UCEC (Uterine Corpus Endometrial Carcinoma) (**Figure 1A**).

Because data on normal tissue distribution was not available for some tumor types in TCGA, we next analyzed differential expression of LIFR between normal and tumor tissues *via* the GTEx database. Downregulated expression of LIFR in tumor tissues was seen in ACC (Adrenocortical carcinoma), LAML (Acute Myeloid Leukemia), OV (Ovarian serous cystadenocarcinoma), TGCT (Testicular Germ Cell Tumors), and UCS (Uterine Carcinosarcoma), while there was a significant upregulation in LGG (Brain Lower Grade Glioma) and THYM (Thymoma) (**Figure 1B**). For other tumors, such as DLBC (Lymphoid Neoplasm Diffuse Large B-cell Lymphoma) and SARC (Sarcoma), there were no significant differences (**Figure S1C**). Overall, LIFR was significantly more weakly expressed in multiple cancers than in the corresponding normal tissues.

We also assessed LIFR expression at the protein level and found significant downregulation in BLCA, BRCA, CHOL, COAD, ESCA, HNSC, KICH, KIRC, KIRP, LIHC, LUAD, LUSC, PRAD, READ, STAD, THCA and UCEC tumor tissues (**Figure 1C**).

Finally, we sought correlations between LIFR expression and tumor pathological staging, which suggested stage-specific alternations in LIFR for a few tumors, such as CESC, KIRC and THCA (**Figure 1D**), while in most tumor types we found no significant correlations (**Figure S2**).

### Prognostic value of LIFR

**Figure 2:**
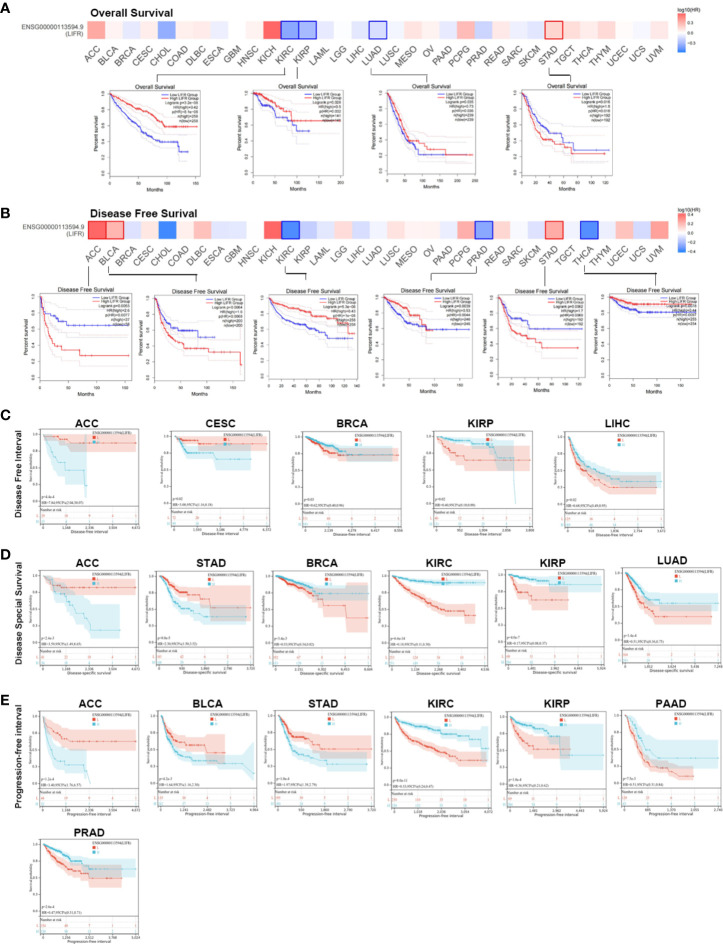
Association between LIFR expression level and survival in TCGA. **(A)** Association between LIFR gene expression and overall survival. High level LIFR resulted in better OS for tumors of KIRC, KIRP, and LUAD, but resulted in worse OS for STAD. **(B)** Association between LIFR gene expression and disease-free survival. High expression of LIFR was related to worse prognosis for tumors of ACC, BLCA and STAD. LIFR was a protective factor for KIRC, PRAD and THCA. **(C)** Association between LIFR gene expression and disease-free interval. Patients with low LIFR had better DFI in ACC, CEAC, while in patients with BRCA, KIRP and LIHC, low LIFR expression was related to poor DFI. **(D)** Association between LIFR gene expression and disease-special survival. Low LIFR was significantly associated with better DSS in ACC and PAAD, but the high LIFR expression induced a better DSS for patients with BRCA, KIRC, KIRP, LUAD and SKCM-M. **(E)** Association between LIFR gene expression and progression-free interval. Low LIFR was significantly associated with better PFI in ACC, BLCA, and STAD, but the high LIFR expression induced a better PFI for patients with KIRP, PAAD and PRAD.

We investigated the prognostic value of LIFR and found that higher levels correlated with better overall survival (OS) for tumors of KIRC, KIRP, and LUAD within the TCGA datasets, but the opposite result for STAD(**Figure 2A**). Analysis of disease-free survival (DFS) showed a correlation between highly expressed LIFR and a worse prognosis for ACC, BLCA and STAD, although it was a protective factor for KIRC, PRAD and THCA (**Figure 2B**).

**Figure 3:**
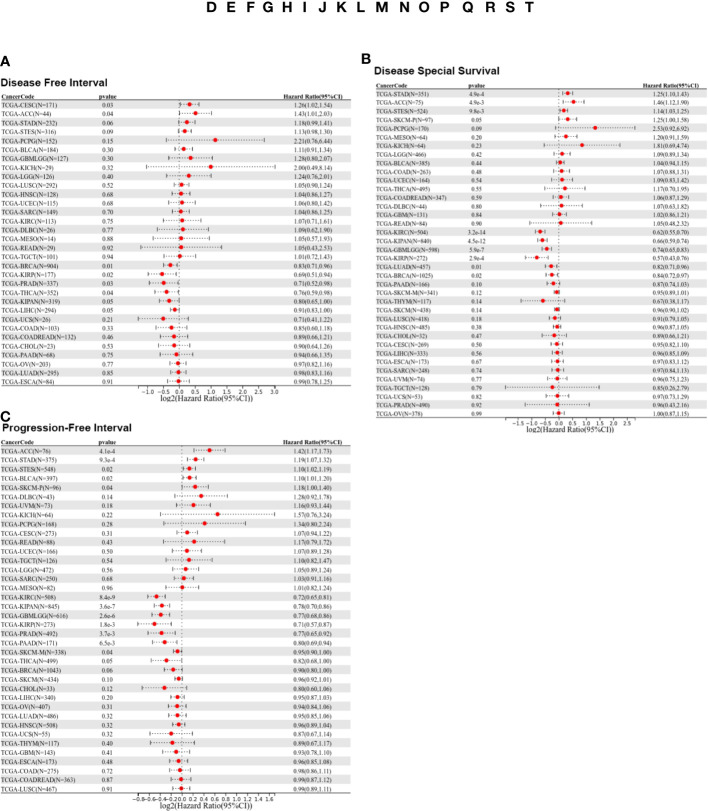
Prognostic analysis of LIFR *via* multivariable Cox regression. **(A)** The COX proportional hazards analysis indicated that high LIFR expression led to a poor DFI in CESC and ACC. On the contrary, elevated LIFR resulted in better DFI in BRCA, KIRP, PRAD, THCA and LIHC. **(B)** The COX proportional hazards analysis indicated that higher LIFR was significantly related to a poorer DSS in STAD and ACC. In contrast, low LIFR expressed patients had poorer DSS in KIRC, KIRP, LUAD and BRCA. **(C)** The COX proportional hazards analysis indicated that increased expression of LIFR was related to a poorer PFI of patients with ACC, STAD, and BLCA, however, in patients with KIRC, KIRP, PRAD and THCA, LIFR expression exhibited the opposite correlation with prognosis.

To further investigate the prognostic potential of LIFR, we analyzed some other prognostic indicators, including disease-specific survival (DSS), disease-free interval (DFI), and progression-free interval (PFI) *via* the Kaplan–Meier method and univariate Cox regression. Cox proportional hazards analysis indicated that high LIFR expression led to a poor DFI in CESC and ACC. On the contrary, elevated LIFR resulted in better DFI in BRCA, KIRP, PRAD, THCA and LIHC (**Figure 3A**). Patients with low LIFR had better DFI in ACC, CEAC, while in patients with BRCA, KIRP and LIHC, low LIFR expression was related to poorer DFI (**Figure 2C**).

Moreover, higher LIFR was significantly related to a poorer DSS in STAD and ACC. In contrast, low LIFR corelated with poorer DSS in KIRC, KIRP, LUAD and BRCA (**Figure 3B**). Kaplan-Meier analysis indicated that low LIFR was significantly associated with better DSS in ACC and PAAD, but high LIFR expression correlated with a better DSS for patients with BRCA, KIRC, KIRP, LUAD and SKCM-M (**Figure 2D**).

Regarding associations between LIFR expression and PFI, the results demonstrated that higher expression of LIFR was related to a poorer PFI of patients with ACC, STAD, and BLCA, but in patients with KIRC, KIRP, PRAD and THCA, LIFR expression exhibited the opposite correlation with prognosis (**Figure 3C**). Kaplan–Meier survival curves with were shown in **Figure 2E**. These data suggested that LIFR was an independent prognostic marker for many cancers.

### Genetic alternation analysis of LIFR

**Figure 4:**
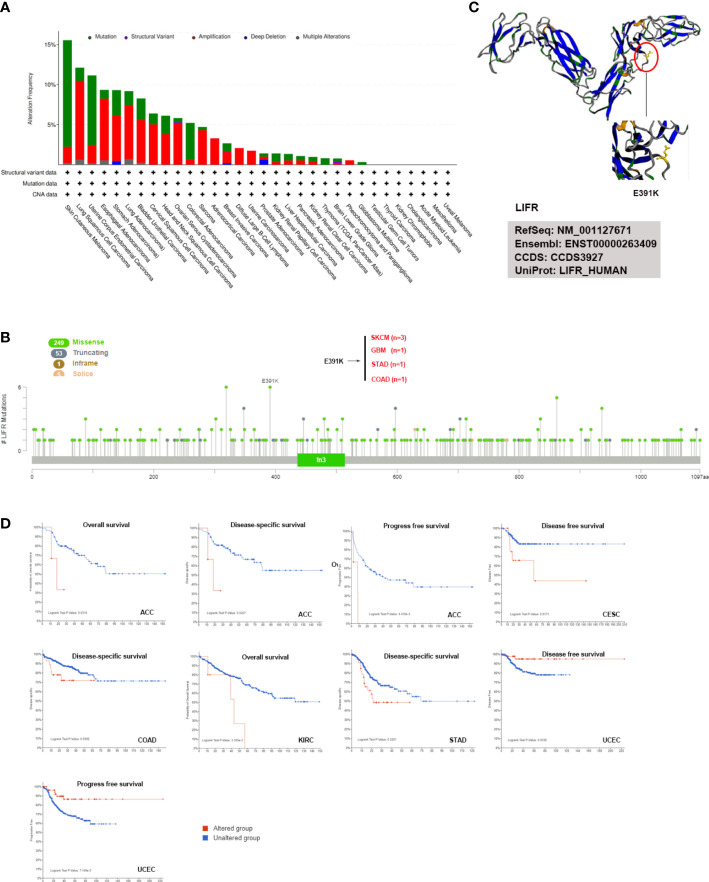
LIFR mutation features in different tumor types of TCGA. **(A)** Analysis of LIFR alteration frequency in different tumor types according to cBioPortal dataset. The highest LIFR alteration frequency (>13%) was observed for patient with SKCM with “mutation”. DLBC had the highest incidence of “amplification” (>9%) of copy number alteration (CNA). **(B)** The mutational landscape and the mutation site of LIFR in pan-cancer analysis according to cBioPortal dataset. Missense mutation of LIFR was the main genetic alternation type, and E391K alternation was detected in 3 cases of SKCM, 1 case of GBM, 1 case of STAD and 1 case of COAD. **(C)** Mutation site with the highest alteration frequency (E391K) was shown in the 3D structure of LIFR. **(D)** Relationship between LIFR mutation status and survival in ACC, CESC, COAD, KIRC, STAD and UCEC using the cBioPortal tool.

The accumulation of genetic alternations influences human cancer development. Thus, the genetic alternations of LIFR were investigated in various human cancer samples. **Figure 4A** showed that the highest frequency of LIFR alternation (>13%) was observed in SKCM for “mutation”, whereas DLBC exhibited the highest incidence of “amplification” (>9%) of copy number alteration (CNA). The types, case numbers and location of LIFR genetic alternations were depicted in **Figure 3B**. Missense mutation of LIFR was the main type of genetic alternation, with E391K detected in 3 cases of SKCM, 1 case of GBM, 1 case of STAD and 1 case of COAD (**Figure 4B**). This mutation induced a frame shift E (Glutamic acid) to K (Lysine) at the position 391 of LIFR protein. Next, we examined the 3D structure of the LIFR protein with this mutation (**Figure 4C**). Further, we investigated potential correlations between genetic alternations to LIFR and the clinical prognosis of cancers (**Figure 4D**). ACC cases with LIFR alternations had a poor prognosis in terms of OS, DSS and PFS (progression free survival). LIFR alternations were also correlated with poor DSS in CESC, with poor DSS in COAD, with poor OS in KIRC, and with poor DSS in STAD. However, LIFR alternations correlated with better DFS and PFS in UCEC. TMB (Tumor mutational burden) and MSI (Microsatellite instability) are crucial factors reflecting prognosis and immune response. Correlations between LIFR expression and TMB and MSI may be a potential biomarker for clinical immunotherapy response in patients with tumors with different LIFR expression patterns. As presented in **Figure S3**, we found strong correlations between LIFR expression and TMB and MSI in most cancer types (**Figures S3A, B**). We then explored the correlation of LIFR expression with genetic alterations in various tumor types. For Simple Nucleotide Variation (SNV), we found no significant correlation in most tumor types, with the exception of UCEC (**Figure S3C**). In terms of Copy Number Variation (CNV), we detected correlations of LIFR expression with genetic alterations for a few tumors, such as LGG, LUAD, BRCA, SARC, PRAD, MESO, OV, and BLCA (**Figure S3D**).

### DNA methylation analysis of LIFR

**Figure 5:**
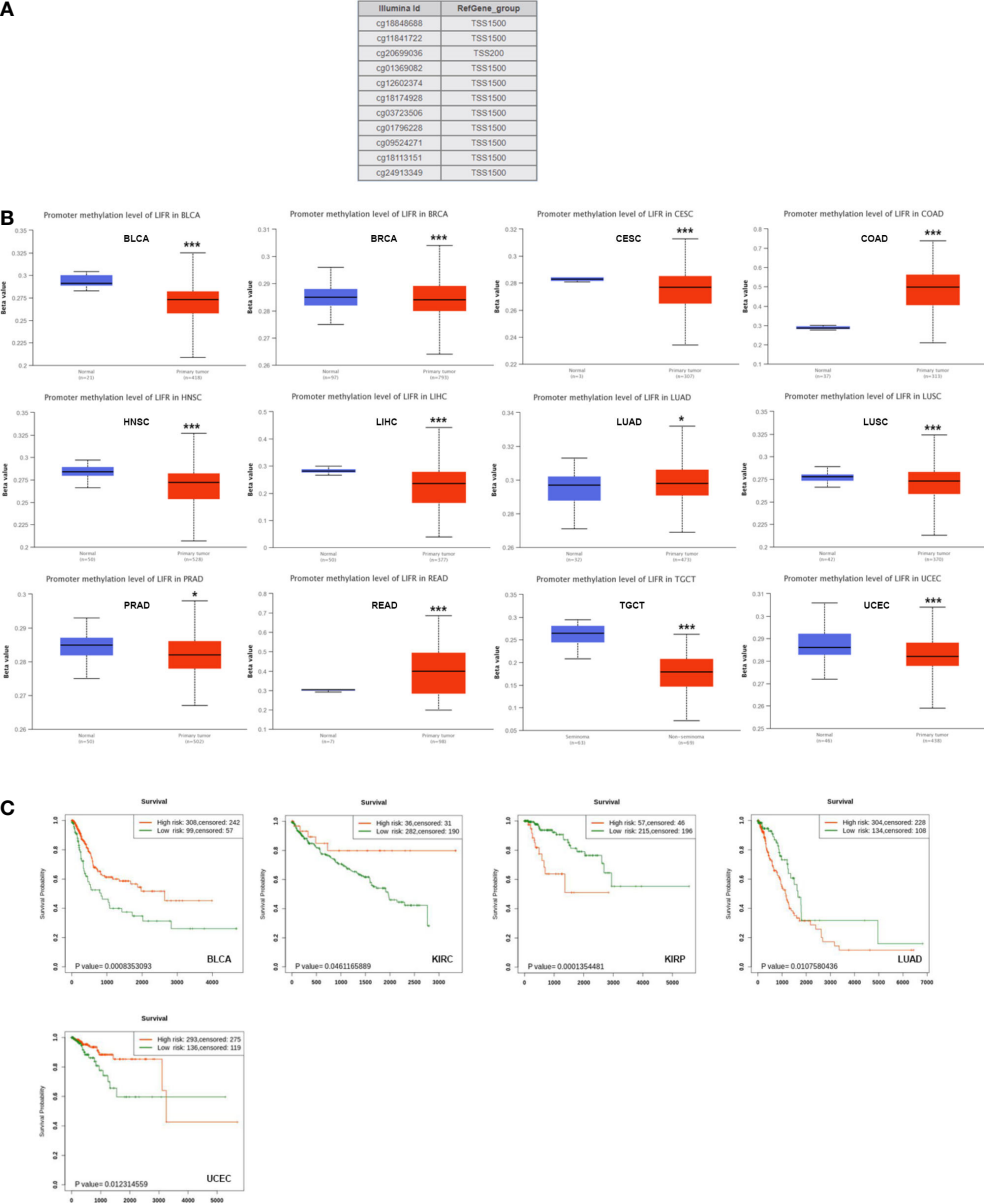
DNA methylation level of LIFR in tumors. **(A)** Probes for detecting DNA methylation of LIFR promoter in UALCAN database. **(B)** DNA methylation level of LIFR in BLCA, BRCA, CESC, COAD, HNSC, LIHC, LUAD, LUSC, PRAD, READ, TGCT and UCEC. DNA promotor methylation level of LIFR was decreased in BLCA, BRCA, CESC, HNSC, LIHC, LUSC, PRAD, TGCT and UCEC tumor tissues. The methylation level of LIFR DNA promotor was increased in COAD, LUAD and READ tumor tissues according to the UALCAN database. *p <.01; ***p <.001. **(C)** Relationship between LIFR DNA methylation level and survival in BLCA, KIRC, KIRP, LUAD and UCEC. LIFR methylation was a protective factor for BLCA, KIRC and UCEC. Low LIFR methylation level resulted in poor prognosis for KIRP and LUAD.

DNA methylation directly affects cancer occurrence and progression. 11 probes were used to explore DNA methylation of LIFR (**Figure 5A**). We observed decreased DNA promotor methylation of LIFR in BLCA, BRCA, CESC, HNSC, LIHC, LUSC, PRAD, TGCT and UCEC tumor tissues. In contrast, significantly increased methylation of LIFR DNA promotor was observed in COAD, LUAD and READ tumor tissues according to the UALCAN database (**Figure 5B**). Further, we Further, we applied Kaplan-Meier survival analysis to explore relationships between LIFR methylation and patient prognosis (22). LIFR methylation was a protective factor for BLCA, KIRC and UCEC. Moreover, we observed a correlation between low LIFR methylation level and poor prognosis for KIRP and LUAD (**Figure 5C**). We then examined the relationship between DNA methylation of LIFR and LIFR expression. LIFR methylation correlated significantly with gene expression at multiple probes in many cancer types (**Figure S4**). These data indicated that the DNA methylation might be not the only reason of abnormal LIFR expression. Other possibilities may contribute to the abnormal expression of LIFR, which need further exploration.

### The correlation between LIFR and tumor purity, immunoregulation associated genes and immune checkpoints

We quantified tumor purity based on the estimation score, which was used to estimate stomal and immune components in tumor tissues. LIFR was significant positively related to the estimate score in several tumors, such as ESCA, COAD, STAD, HNSC, LIHC, SKCM, BLCA, READ, PAAD, DLBC and CHOL, but there was a negative correlation in GBM, UCEC, KIRP, THCA, ACC (**Figure S5**). Regarding the immune score, there was a significantly positive correlation in ESCA, COAD, STAD, HNSC, BLCA, READ, PAAD and CHOL, while there was a clear negative correlation in GBM, UCEC, KIRP, THCA and ACC (**Figure S6**). Additionally, LIFR was positively correlated with the stromal score in BRCA, ESCA, COAD, STAD, HNSC, LIHC, SKCM, BLCA, READ, PAAD, USC, LAML and CHOL (**Figure S7**). These results indicated that elevated LIFR was usually accompanied by immune infiltration in the tumor microenvironment.

**Figure 6:**
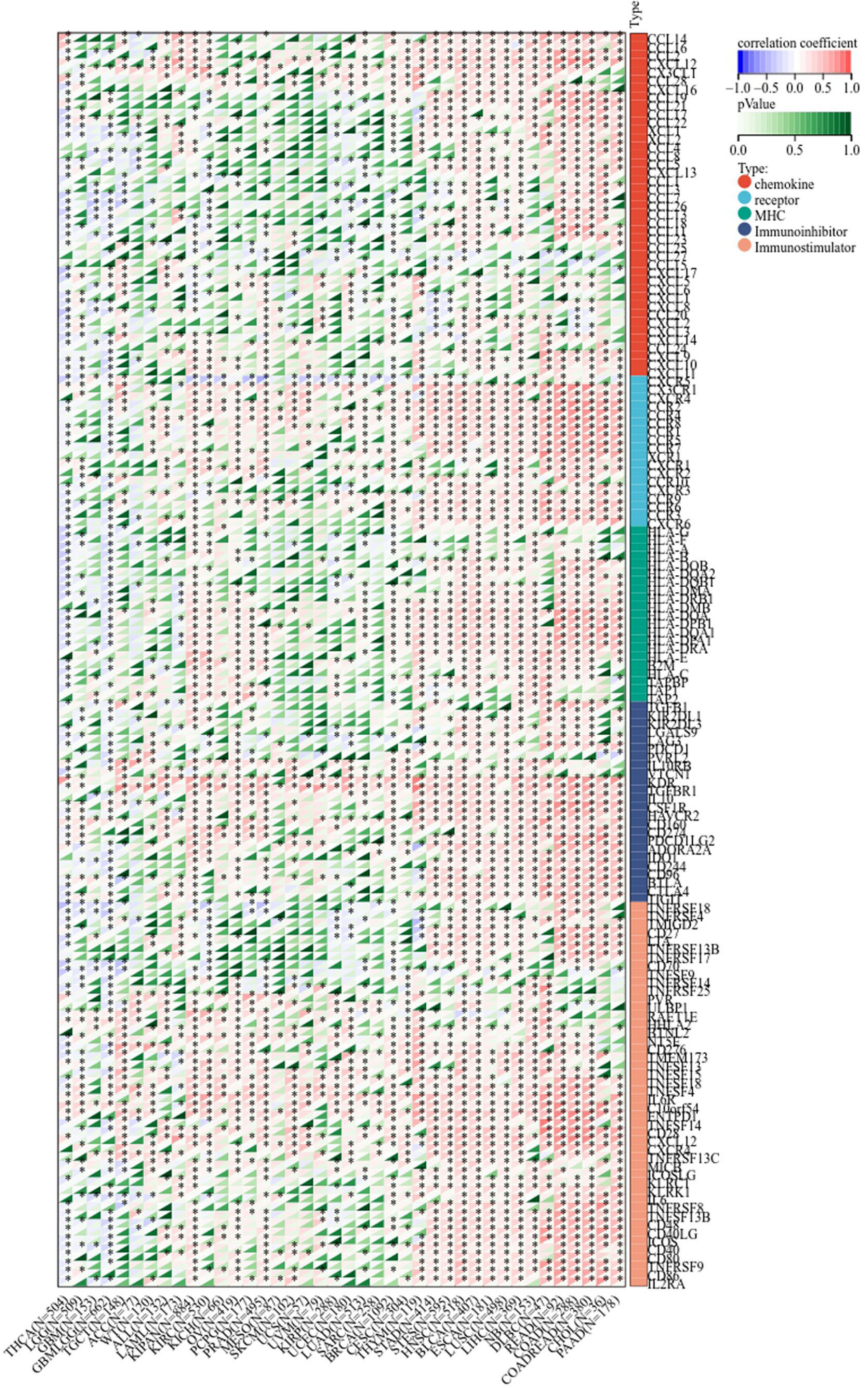
Co-expression of LIFR and immune-related genes including chemokines, chemokine receptors, MHC, immunoihibitors, and immunostimulators. LIFR was obviously correlated with immunoregulation related genes in most cancer types. *P < 0.05.

**Figure 7:**
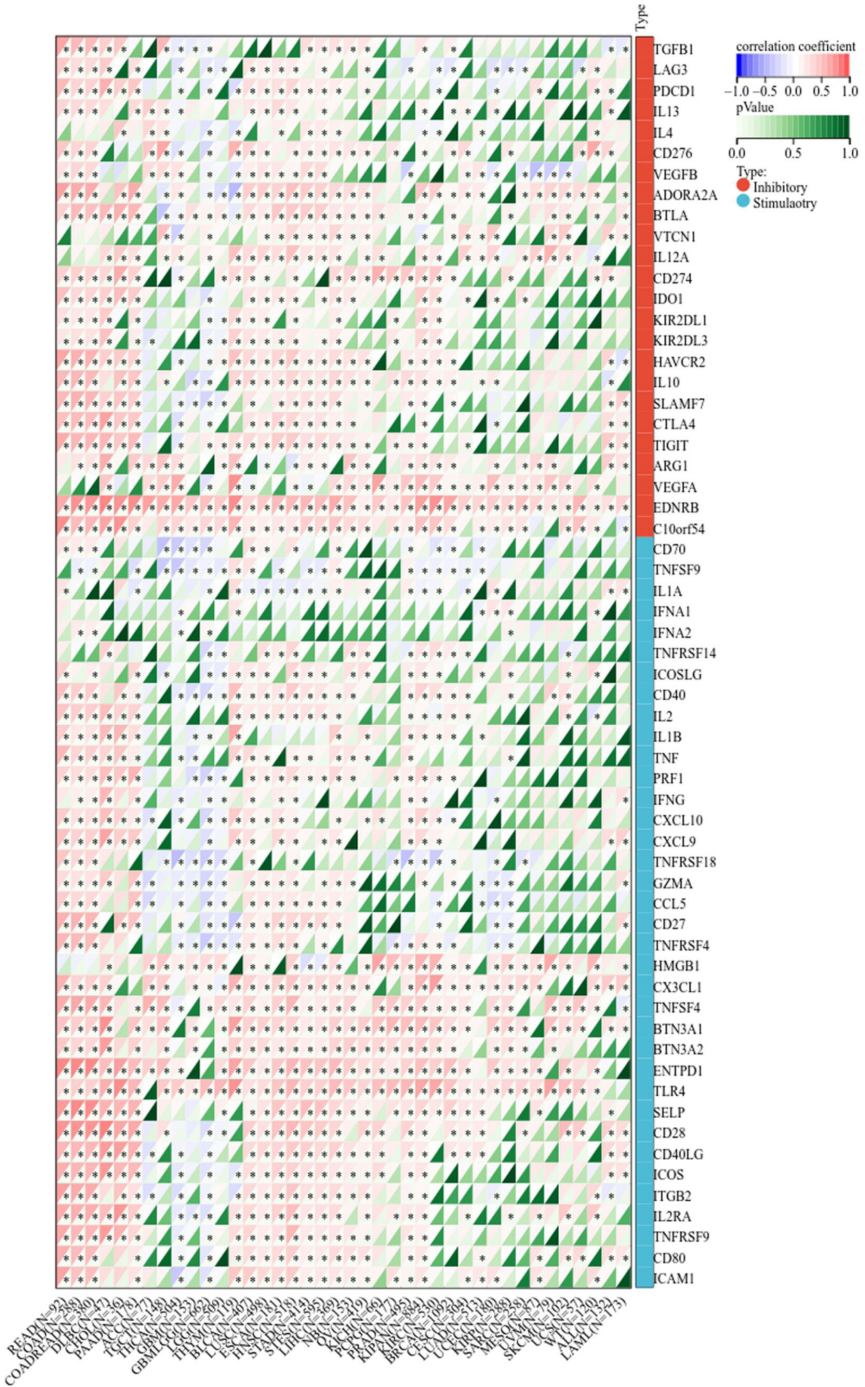
Correlation between LIFR expression and immune checkpoint genes in different tumor types. In most tumors, except LAML, UCS, SKCM, UVM, MESO and SARC, significant correlations existed between the expression of LIFR and recognized immune checkpoints such as EDNRB, C10orf54, HMGB1, CX3CL1, TNFSF4, BTN3A1, ENTPD1 and TLR4. *P < 0.05.

Next, we determined the relationship between LIFR and immunoregulation-related genes, and saw clear correlations in most cancer types (**Figure 6**). The correlation between LIFR and immune checkpoints was shown as **Figure 7**. In most tumors, except LAML, UCS, SKCM, UVM, MESO and SARC, significant correlations were present between the expression of LIFR and recognized immune checkpoints such as EDNRB (Endothelin receptor type B), C10orf54 (V-type immunoglobulin domain-containing suppressor of T-cell activation), HMGB1 (High mobility group protein B1), CX3CL1, TNFSF4 (Tumor necrosis factor ligand superfamily member 4), BTN3A1 (Butyrophilin subfamily 3 member A1), ENTPD1 (Ectonucleoside triphosphate diphosphohydrolase 1) and TLR4 (toll-like receptor 4), suggesting a potential correlation between LIFR and known immune checkpoints. Notably, LIFR was positively correlated with EDNRB in all tumors (**Figure 7**).

### Immune infiltration analysis

**Figure 8:**
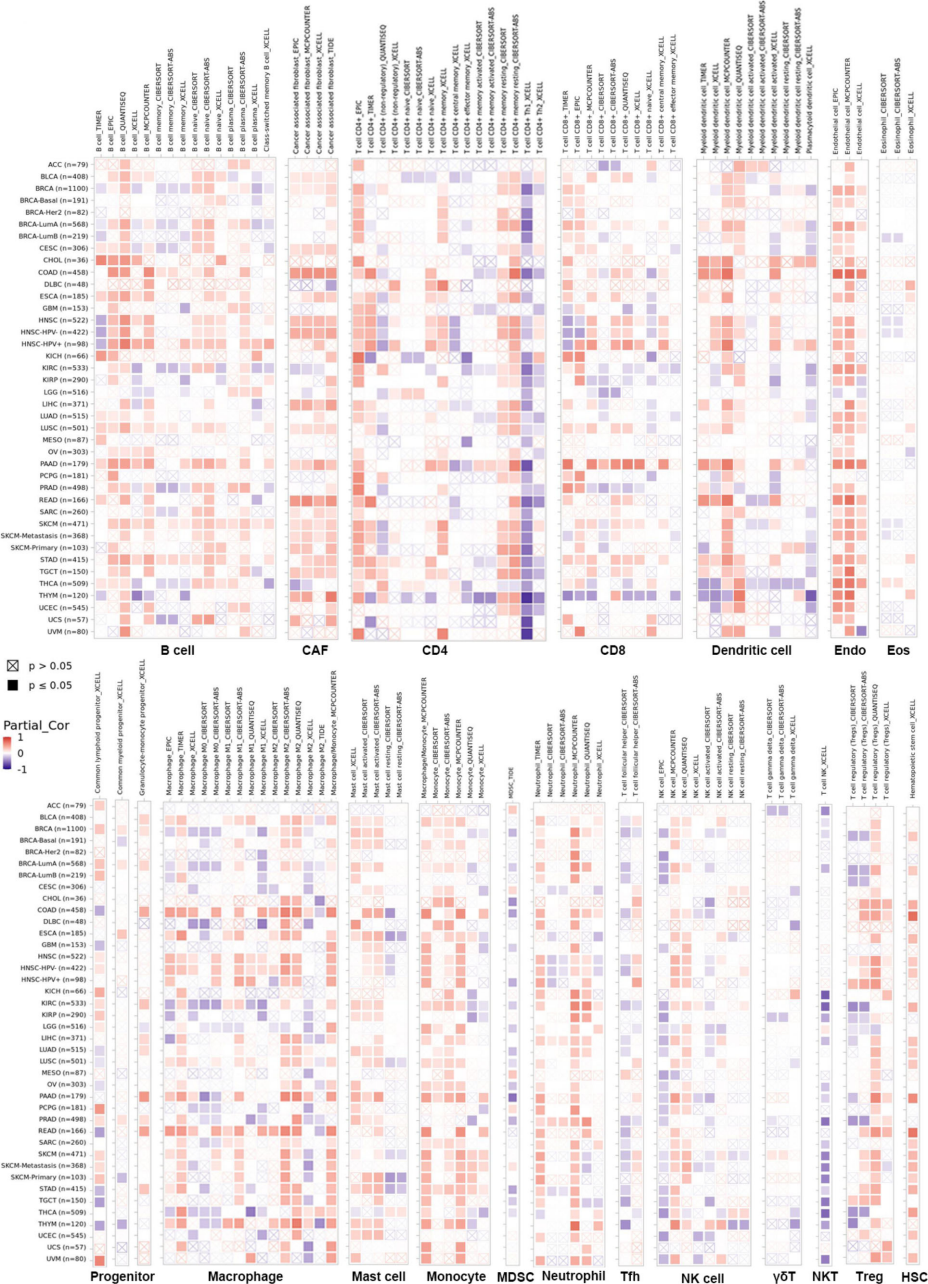
The correlation between LIFR expression and immune infiltration across all tumor types in TCGA. Positive correlation (0–1) in red and negative correlation (−1–0) in blue. P-value <0.05 is considered as statistically significant. Statistically non-significant correlations values as P-value >0.05 are marked with a cross. LIFR was positively related to many kinds of immune infiltrating cells including endothelial cells, cancer-associated fibroblast, monocyte, mast cells and hematopoietic stem cells. However, LIFR was negatively correlated with natural killer T cells (NKT) abundance.

The infiltration of immune cells in tumors intimately affects their initiation, progression and metastasis (23, 24). Therefore, we sought a correlation between LIFR expression and various immune infiltrates across human cancers (**Figure 8**). Overall, LIFR was positively related to many kinds of cells in the microenvironment, including endothelial cells, cancer-associated fibroblasts, monocytes, mast cells and hematopoietic stem cells. However, LIFR was negatively correlated with natural killer T cell (NKT) abundance. This profile suggested that LIFR has a critical role in immune-oncological interactions. Interestingly, in some tumors, the trend of the relationship was subtly different because of different immune infiltration ratios in various tumors.

### Enrichment analysis of LIFR-related partners

**Figure 9:**
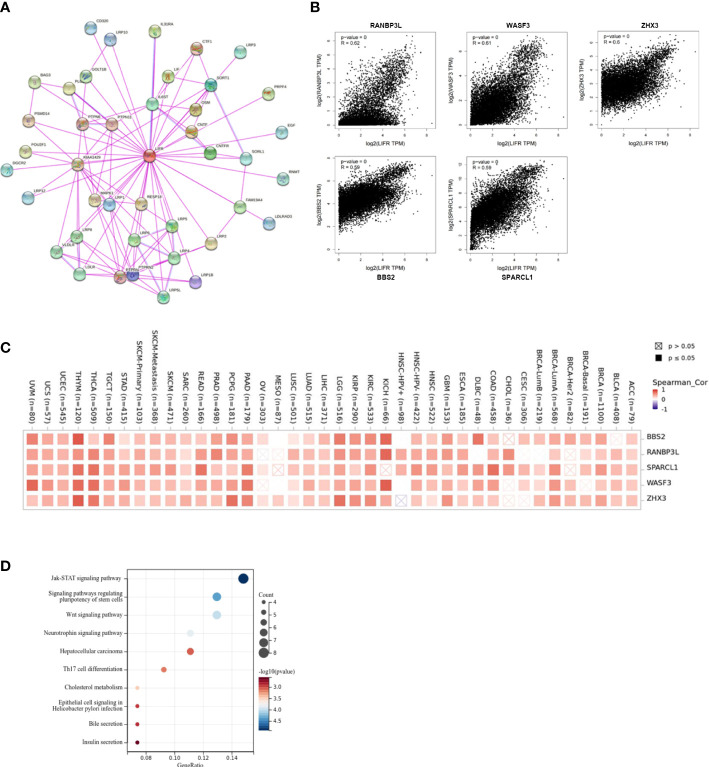
Enrichment and functional analysis of LIFR-related gene. **(A)** STRING protein network presents the proteins interacting with LIFR. **(B)** Expression relationship between LIFR and representative top LIFR-correlated genes including BBS2, RANBP3L, SPARCL1, WASF3 and ZHX3 in TCGA as determined by GEPIA2. **(C)** Heatmap representation of the expression correlation between LIFR and BBS2, RANBP3L, SPARCL1, WASF3 and ZHX3 in TCGA tumors. **(D)** Functional analysis based on the combination of LIFR-binding and interacting genes.

To identify the mechanism of LIFR action in tumorigenesis and cancer progression, we explored targeting LIFR-interacting proteins and LIFR-expression related genes and performed functional enrichment analysis. We identified several experimentally detected LIFR-binding proteins from the STRING dataset. The interaction network was shown in **Figure 9A**. Based on the GEPIA2 tool, we acquired genes related to LIFR expression. In **Figure 9B**, LIFR was seen to be positively correlated with BBS2 (Bardet-Biedl syndrome 2 protein), RANBP3L (Ran-binding protein 3-like), SPARCL1 (SPARC-like protein 1), WASF3 (Actin-binding protein WASF3) and ZHX3 (Zinc fingers and homeoboxes protein 3) (**Figure 9B**). The heatmap showed positive correlations in most tumor types (**Figure 9C**). We then conducted functional enrichment analyses by combining the two datasets. The results suggested that “JAK-STAT signaling pathway”, “signaling pathways regulating pluripotency of stem cells” and “Wnt signaling pathway” might be related to the tumor pathogenesis function of LIFR (**Figure 9D**).

### The expression and functional role of LIFR in UCEC

**Figure 10:**
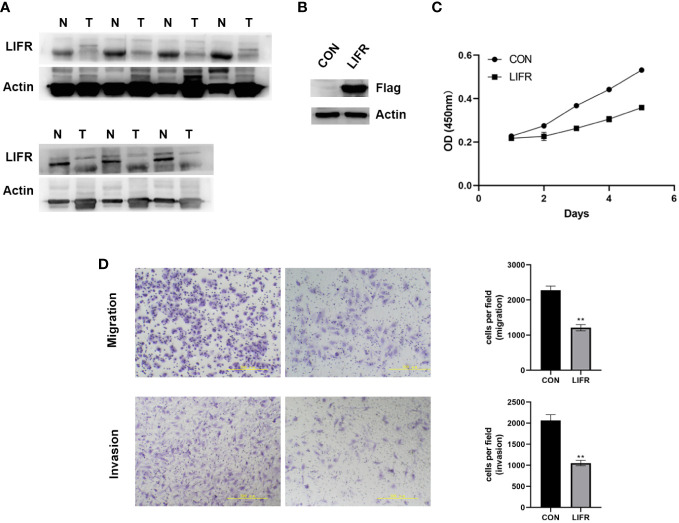
The Expression and Functional Role of LIFR in UCEC. **(A)** The protein expression levels of LIFR in paired UCEC and adjacent normal tissues by western blot. LIFR was low expressed in tumor tissues. **(B)** Overexpressed the expression of LIFR in human UCEC cell line Ishikawa and the efficiency of LIFR expression was confirmed by western blot. **(C)** CCK-8 assay results showing the decrease in the viability of Ishikawa cells upon the overexpression of LIFR. **(D)** Transwell migration/invasion assays upon LIFR overexpression in Ishikawa. LIFR expression inhibited the migration/invasion ability of Ishikawa. **P < 0.01.

To clarify whether LIFR is an anti-tumorigenic factor in UCEC, we investigated its expression in 7 pairs of UCEC clinical samples by Western blot. LIFR was expressed at a low level in UCEC tumor tissues (**Figure 10A**). We overexpressed LIFR in the human UCEC cell line Ishikawa and the efficiency of LIFR expression was confirmed as shown in **Figure 10B**. CCK8 assay showed that the overexpression of LIFR decreased the viability of Ishikawa cells *in vitro* (**Figure 10C**).

​To determine whether LIFR is a functional gene in UCEC cells, we estimated cancer cell migrative and invasive abilities by Trans-well migration/invasion assays. We investigated whether LIFR overexpression repressed the migrative and invasive capacities of Ishikawa. The migration and invasion of UCEC cells were remarkably suppressed upon LIFR overexpression in Ishikawa (**Figure 10D**), indicating that LIFR represses the progression and aggression of UCEC.

## Discussion

Cancers are the second major cause of death worldwide (25), so an improved understanding of how they arise and how to treat them more effectively remains a pressing need. Although increasing numbers of studies have investigated the expression and functions of LIF within various cancers, there are still have limitation of clinical trials that explore a therapeutic agents aimed at affecting LIF signaling for improving outcomes for patients with cancers.

As a pleiotropic glycoprotein, LIF belongs to the IL-6 cytokine family and is highly conserved across species. The interaction of LIF-LIFR triggers multiple signaling pathways, such as AKT, mTOR and STAT3 (9, 26), thus providing an impetus to tumor cell EMT, migration and invasion, both *in vitro* and *in vivo*. LIF mediates signaling *via* membrane receptor complex comprised of gp130 and LIFR (27). LIF-LIFR signaling is involved in cancer progression and its deregulation occurs in multiple cancers. Studies have confirmed the oncogenic functions of LIF-LIFR in tumor stemness, progression, alterations in the tumor microenvironment and therapy resistance (18, 26, 28).

Increasingly, the focus has been on the functional exploration of LIFR in disease, but whether LIFR is correlated with the oncogenesis of certain cancer types, or just participates in more common pathways modulating cancer pathogenesis, is still unclear. Thus, we performed a pan-cancer analysis of LIFR. We comprehensively analyzed the profiles of LIFR expression in various tumor types in TCGA database. We also systematically investigated protein data and other genetic alternations and molecular features.

This study explored LIFR expression level and prognostic landscape in pan-cancer. We report that LIFR is more highly expressed in LGG and THYM than the corresponding normal tissues, whereas lower LIFR is present in ACC, BLCA, BRCA, CESC, CHOL, COAD, ESCA, HNSC, KICH, KIRC, KIRP, LAML, LIHC, LUAD, LUSC, OV, PRAD, READ, STAD, TGCT, THCA, UCEC and UCS tumor tissues. Studies have detected the decreased expression level of LIFR and identified the tumor suppressor role of LIFR in different tumor types, such as liver cancer, breast cancer, gallbladder cancer, gastric cancer, lung cancer, pancreatic cancer, clear cell renal cell carcinoma, and colorectal cancer. However, a plethora of evidence has indicated that LIFR performs as a marker of poor prognosis and is highly expressed in several types of tumor tissues, such as melanoma and prostate carcinoma. Based on the results surveyed here, LIFR expression in BRCA tumor tissues is clearly lower than in the corresponding normal tissues. Furthermore, the functional pathway analysis showed the enrichment of “JAK-STAT signaling pathway” among the top hits. LIFR had been previously identified as a suppressor of breast cancer and metastasis (15, 29). Recent studies indicated that the activation of LIFR and downstream STAT3 signaling maintained breast cancer cells in a dormant state and that loss of the LIFR-STAT3 axis led to enhanced proliferation of cancer cells and to bone destruction (30). Furthermore, HDAC inhibitors stimulated LIFR expression in breast cancer cells and reduced proliferation rates (30–32). These results imply a critical role for LIFR in BRCA. Collectively, LIFR served as an anti-tumor gene in multiple human cancers. However, LIFR does not always act as a suppressor in carcinogenetic processes, and its function in each type of cancer should be examined through targeted research. Differences in the expression level of LIFR in different tumors suggest distinct underlying mechanisms of action.

Survival analysis revealed a protective function of LIFR in KIRC. Lei et al. explored the function and mechanism of action of LIFR in clear cell renal cell carcinoma (ccRCC), and found that high LIFR expression predicted a better prognosis and repressed the aggressive tumor phenotype. Moreover, LIFR knockdown promoted the invasion and migration of ccRCC cell lines. Notably, they identified the Hippo pathway as the potential downstream target of LIFR, where LIFR inhibition repressed the kinase activity and upregulated the intracellular Yes-associated protein (YAP) level (33).

We further report that low LIFR expression usually predicted poor OS for patients with KIRC, KIRP and LUAD, but predicted better OS in STAD. For DFS, high LIFR expression resulted poor prognosis in ACC, BLCA, and STAD, but had opposite results in KIRC, PRAD, and THCA. For DFI, low LIFR expression usually predicted better prognosis for patients with ACC and CESC, but predicted poor DFI in BRCA, KIRP, and LIHC. Moreover, higher LIFR was significantly related to a poorer DSS in STAD and ACC. In contrast, low LIFR corelated with poorer DSS in KIRC, KIRP, LUAD and BRCA. Regarding associations between LIFR expression and PFI, the results demonstrated that higher expression of LIFR was related to a poorer PFI of patients with ACC, STAD, and BLCA, but in patients with KIRC, KIRP, PRAD and THCA, LIFR expression exhibited the opposite correlation with prognosis. All these data suggest that LIFR could be a novel biomarker for predicting prognosis.

UCEC tumor immune microenvironment plays an important role in the progression of UCEC. Our results showed that LIFR expression was significantly correlated with the stromal scores in 19 types of cancers, was correlated with the immune scores in 22 types of cancers, and was especially significantly negatively correlated with the immune scores in UCEC. Moreover, we observed that LIFR expression was significantly positively correlated with B cells, CAFs, endothelial cells, mast cells, and neutrophil cells in UCEC.

The bioinformatics data showed the decreased expression of LIFR in tumor tissues compared with normal tissues, and we identified the downregulated expression of LIFR in our collected clinical samples. For genetic alternations of LIFR, the altered group was correlated with better DFS and PFS in UCEC. While according to the data from TCGA, there was no statistically significant correlation between LIFR expression and prognosis for UCEC. We are collecting more clinical samples, and then we will further analyze the expression of LIFR and its relationship with prognosis in the future. We further illustrated the function of LIFR in UCEC by molecular biology methods and demonstrated that the overexpression of LIFR significantly inhibited the proliferation, migration and invasion abilities in UCEC cells. Nonetheless, additional experiments both *in vitro and in vivo* are needed to verify our findings.

In conclusion, our comprehensive pan-cancer survey identified a statistically significant correlation between LIFR expression and prognosis, immune cell infiltration, microsatellite instability, and tumor mutation burden for various cancer types, contributing to clarifying the function of LIFR in tumorigenesis from a variety of perspectives. Our present data identified suppressor effects of LIFR on the progression and migration of UCEC, indicating the potential role of LIFR for predicting patient prognosis and clinical therapy.

## Data availability statement

The original contributions presented in the study are included in the article/**Supplementary Material**. Further inquiries can be directed to the corresponding author.

## Author contributions

FZ and LNT were responsible for experimental design, experimental analysis and thesis writing. YLW, HJL, LL, XFY and XYY were responsible for data screening and collection. FZ and LNT were responsible for the guidance and review of the thesis. All authors contributed to the article and approved the submitted version.
