# Supplementary material for: Pan-cancer analysis identifies LIFR as a prognostic and immunological biomarker for uterine corpus endometrial carcinoma
Source: Front Oncol. 2023 Feb 28;13:1118906. doi: 10.3389/fonc.2023.1118906 (PMC10011451; doi:10.3389/fonc.2023.1118906)

**Supplementary Figure legends**

**Figure S1. The Significantly Differential Expression of LIFR Between Normal and Tumor Tissues.**

**
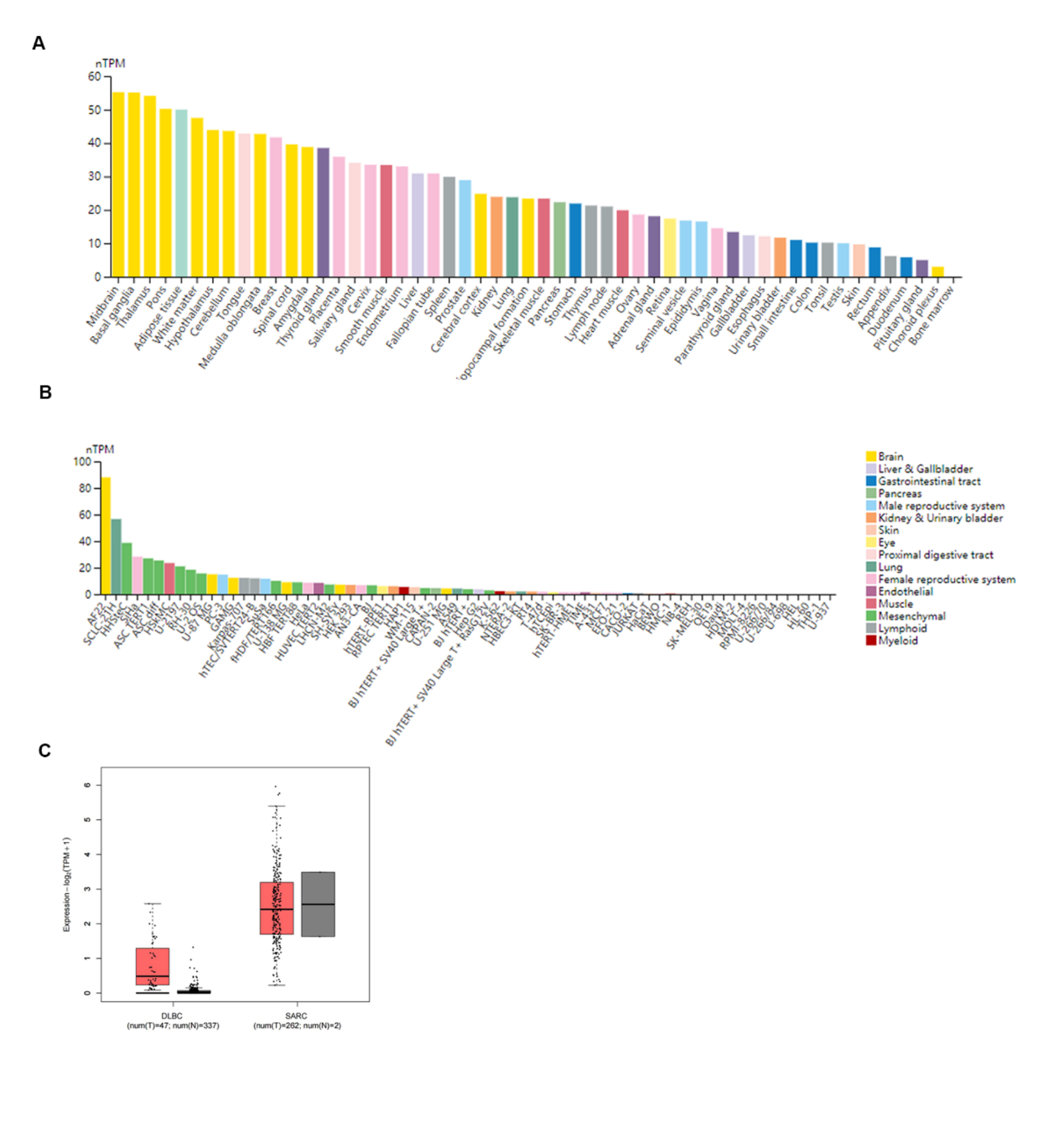
**

**Figure S2**. **Expression level of LIFR by different pathological stages of ACC, BLCA, BRCA, CHOL, COAD, DLBC, ESCA, HNSC, KICH, KIRP, LIHC, LUAD,**

**LUSC, OV, PAAD, READ, SKCM, STAD, TGCT, UCEC and UCS.**

**
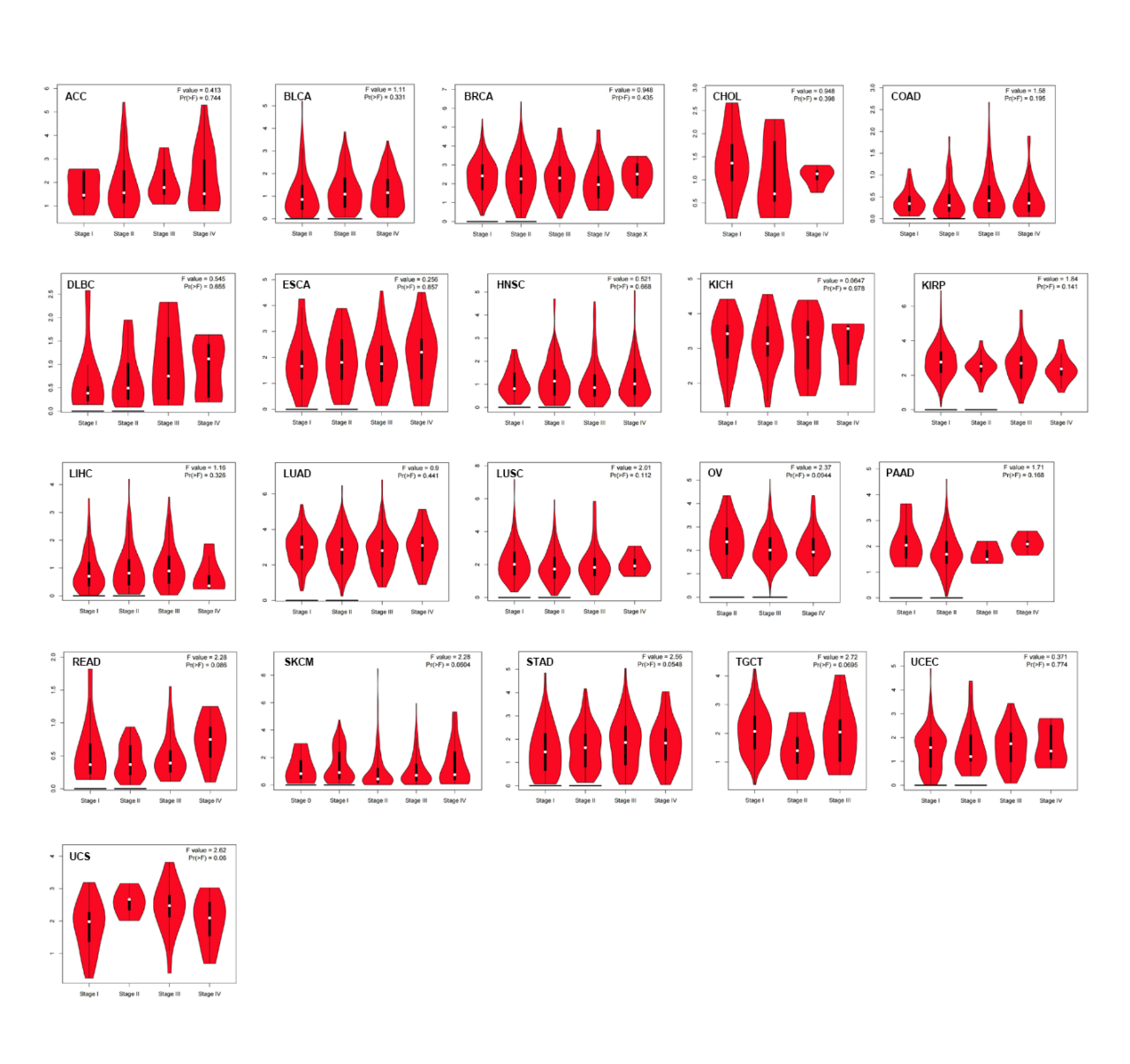
**

**Figure S3. LIFR mutation features in different tumor types**

1. **Correlation between LIFR expression with tumor mutational burden.**
2. **Correlation between LIFR expression with microsatellite instability.**
3. **Correlation between LIFR expression with Simple Nucleotide Variation.**
4. **Correlation between LIFR expression with Copy Number Variation.**


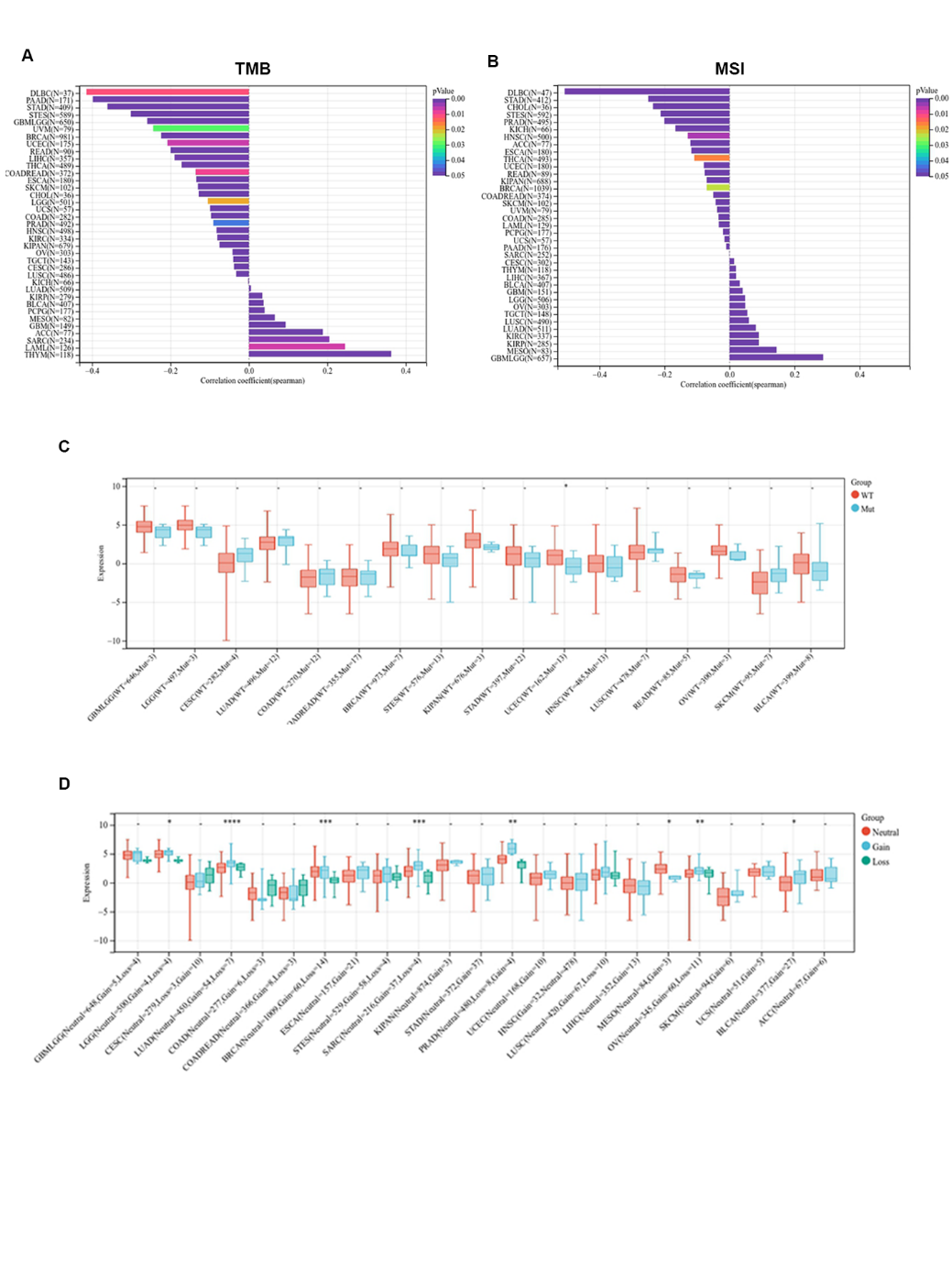


**Figure S4. Correlation between LIFR expression and DNA methylation of LIFR**

**
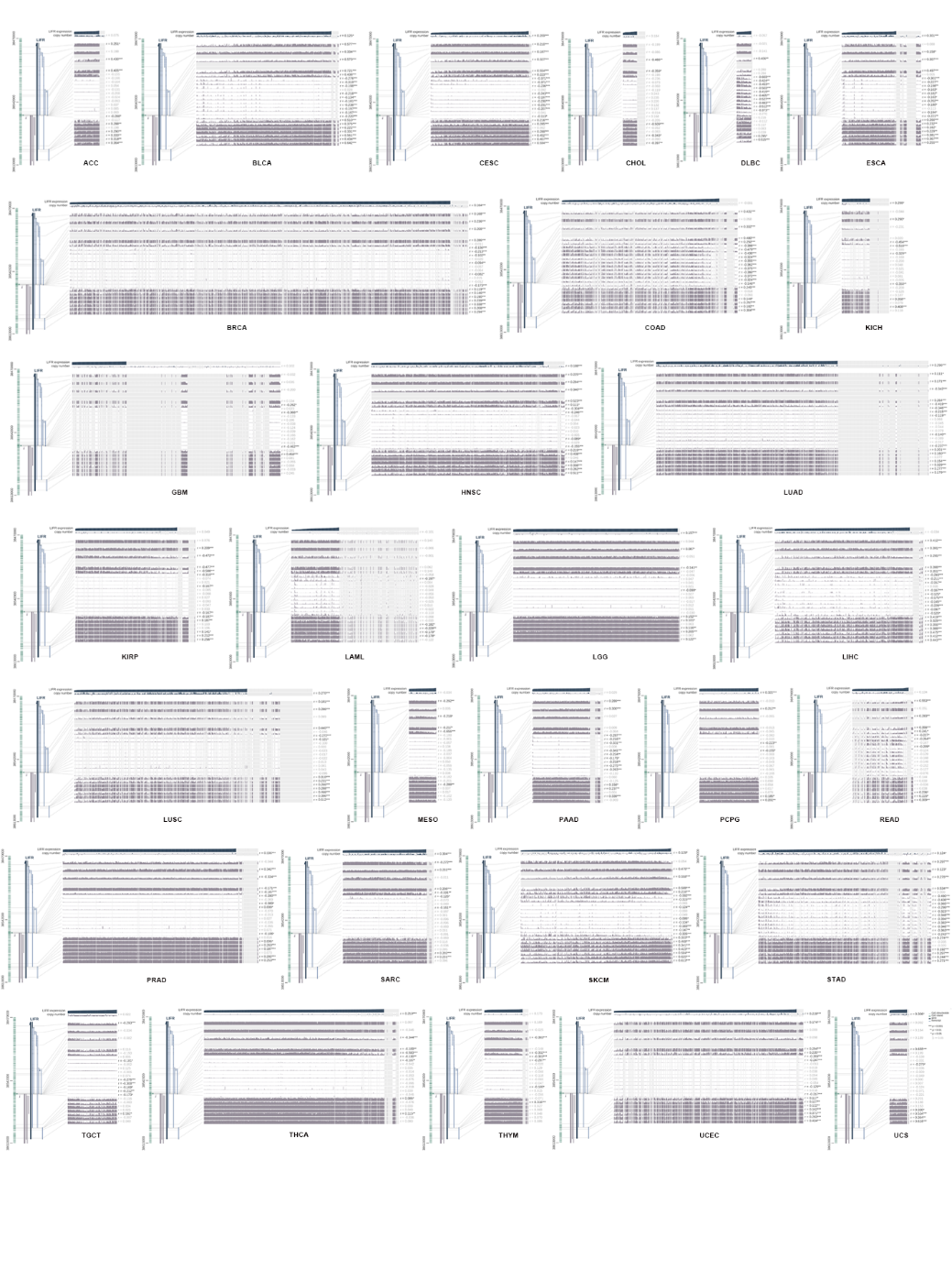
**

**Figure S5. Correlation between LIFR expression and the ESTIMATE score across different cancer types**

**
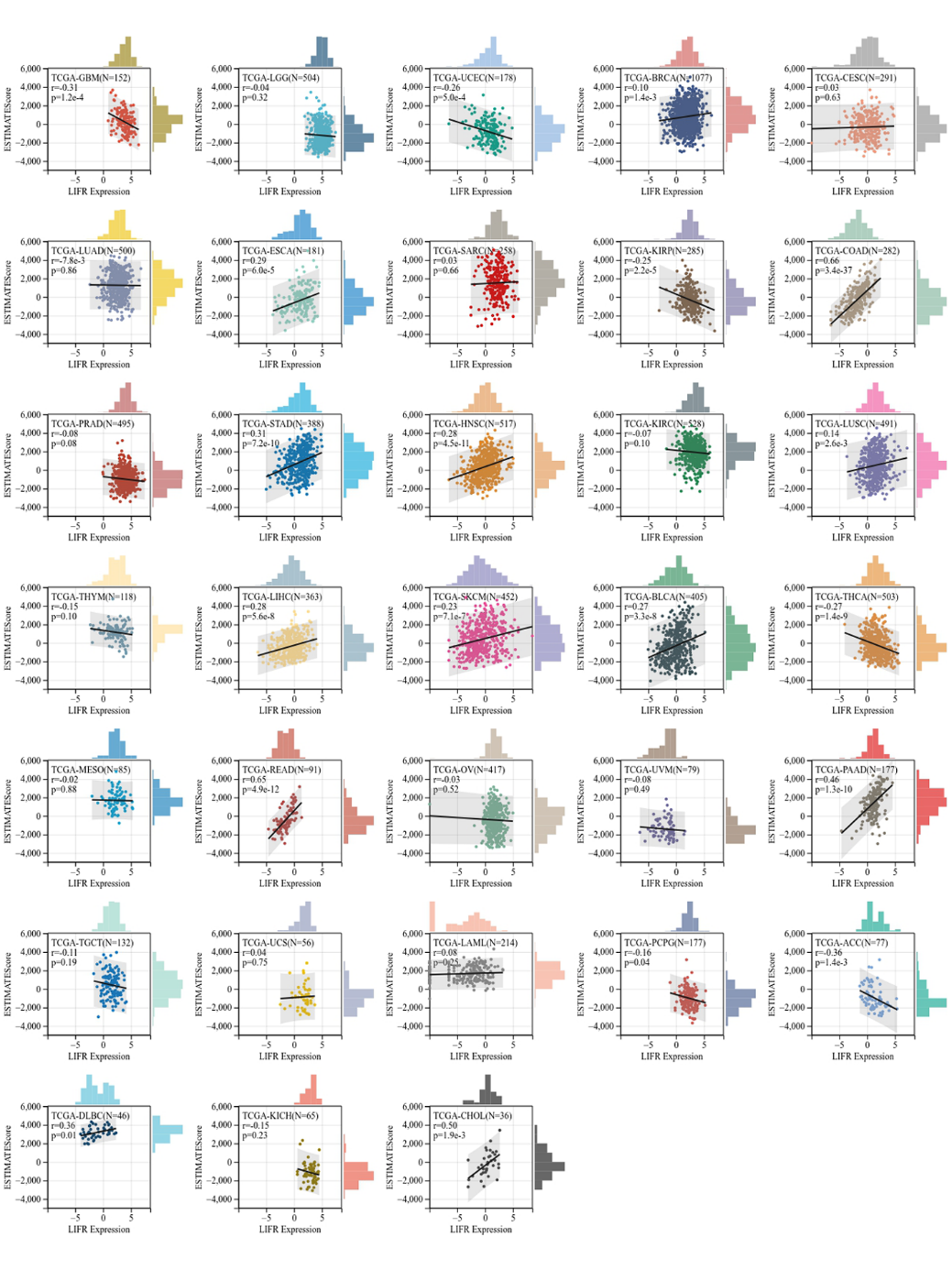
**

**Figure S6. Correlation between LIFR expression and the immune score across different cancer types.**


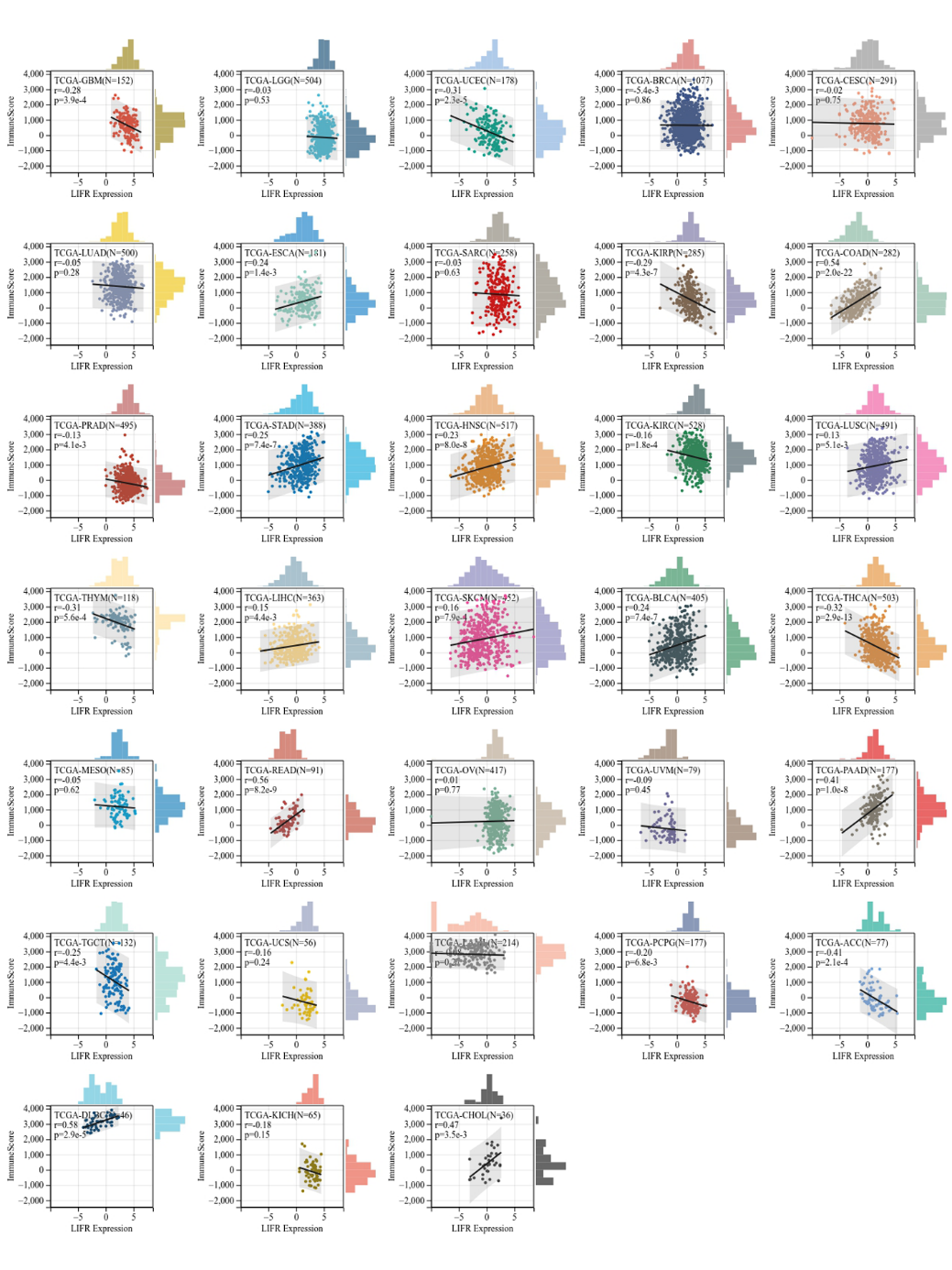


**Figure S7. Correlation between LIFR expression and the stromal score across different cancer types**


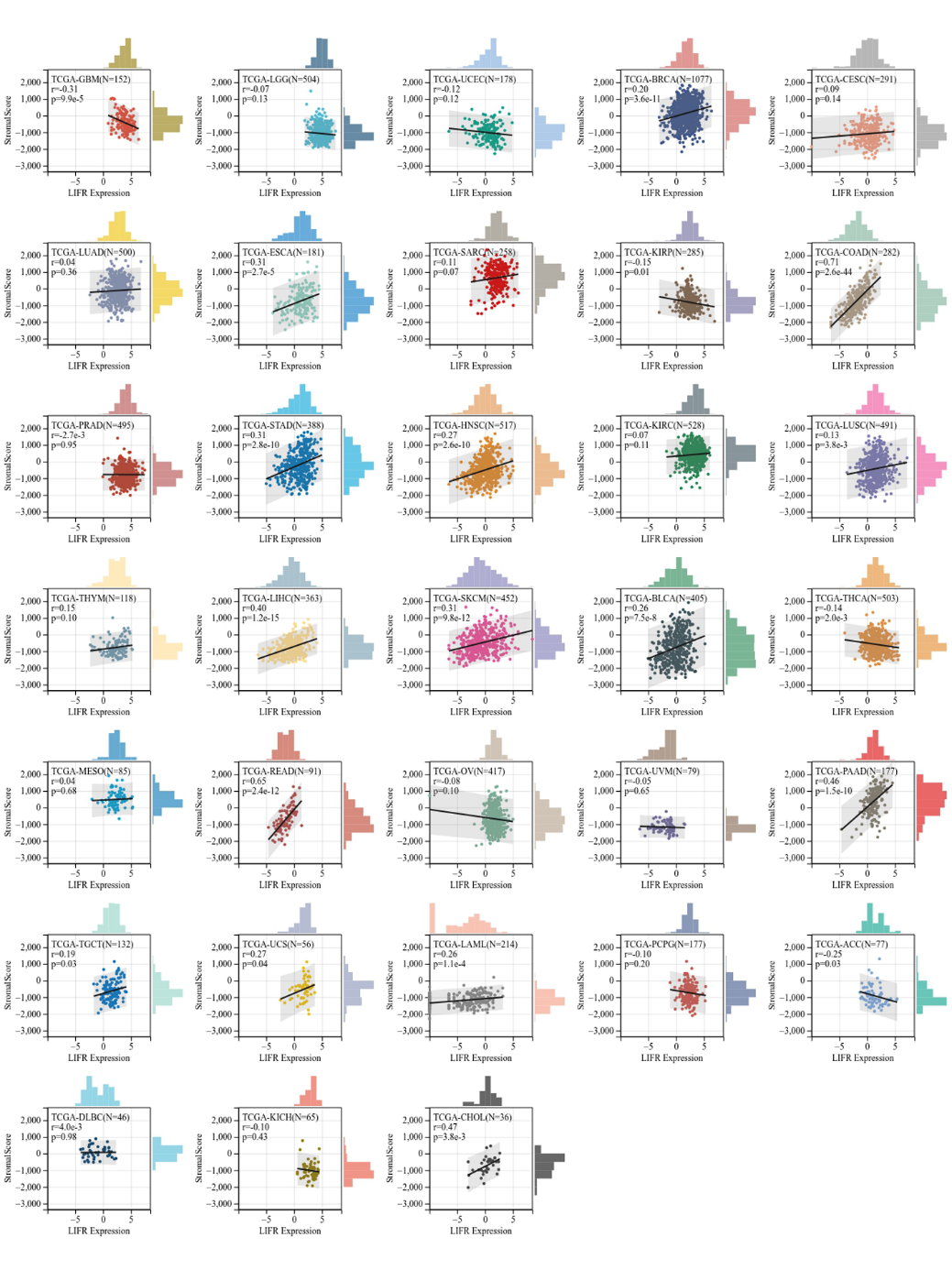

Supplement: Supplementary file 1 [file DataSheet_1.docx]
